# In the shadow of a new smoke free policy: A discourse analysis of health care providers' engagement in tobacco control in community mental health

**DOI:** 10.1186/1752-4458-4-23

**Published:** 2010-07-28

**Authors:** Joy L Johnson, Barbara M Moffat, Leslie A Malchy

**Affiliations:** 1 Nursing and Health Behaviour Research Unit, School of Nursing, University of British Columbia, Vancouver, Canada

## Abstract

**Background:**

The prevalence of tobacco use among individuals with mental illness remains a serious public health concern. Tobacco control has received little attention in community mental health despite the fact that many individuals with mental illness are heavy smokers and experience undue tobacco-related health consequences.

**Methods:**

This qualitative study used methods of discourse analysis to examine the perceptions of health care providers, both professionals and paraprofessionals, in relation to their roles in tobacco control in the community mental health system. Tobacco control is best conceptualised as a suite of policies and practices directed at supporting smoke free premises, smoking cessation counselling and limiting access to tobacco products. The study took place following the establishment of a new policy that restricted tobacco smoking inside all mental health facilities and on their grounds. Ninety one health care providers participated in open-ended interviews in which they described their role in tobacco control. The interview data were analyzed discursively by asking questions such as: what assumptions underlie what is being said about tobacco?

**Results:**

Five separate yet overlapping discursive frames were identified in which providers described their roles. *Managing a smoke free environment *emphasised the need to police and monitor the smoke free environment. *Tobacco is therapeutic *was a discourse that underscored the putative value of smoking for clients. *Tobacco use is an individual choice *located the decision to smoke with individual clients thereby negating a role in tobacco control for providers. *It's someone else's role *was a discourse that placed responsibility for tobacco control with others. Finally, the discourse of *tobacco control as health promotion *located tobacco control in a range of activities that are used to support the health of clients.

**Conclusions:**

This study provides insights into the complex factors that shape tobacco control practices in the mental health field and reinforces the need to see practice change as a matter that extends beyond the individual. The study findings highlight discourses structured by power and powerlessness in environments in which health care providers are both imposing and resisting the smoke free policy.

## Background

The prevalence of tobacco use among individuals with mental illness remains a serious public health concern. Compared to the general population, individuals with mental illness smoke more cigarettes and have greater adverse health outcomes associated with their tobacco use [1]. Tobacco use is also responsible for contributing to economic and social harms for people living with mental illness [2].

The mental health system has not yet developed an appropriate response to tobacco use. Historically, in the mental health field, the role of engaging in smoking cessation intervention has fallen largely to physicians. However, the uptake of these interventions has been limited. In one study, psychiatrists offered cessation counselling during only 12.4% of patients' office visits [3]. The active engagement of health professionals in tobacco cessation interventions for the general adult population seeking medical help is growing and includes brief interventions delivered by counsellors working in substance abuse treatment [4-6]. Yet a similar role for professionals working within community mental health settings is less common [7]. Although one study examined six and 12-month outcomes of a community-based smoking cessation intervention for 79 individuals with severe mental illness, the sessions were facilitated by highly qualified specialists [8] suggesting an expert role without considering similar core skills for a wider group of mental health providers. Of note, these authors reported success rates comparable to group-based treatment with "mentally healthy smokers."

Overall, the mental health system has been slow to implement tobacco cessation interventions [9]. There is, however, strong evidence that many individuals living with mental illness want to reduce or stop smoking altogether [10,11]. Although smoking cessation rates are lower among persons with mental illness than the general population, results are nonetheless substantial [12].

Tobacco control is best conceptualised as a suite of policies and practices directed at supporting smoke free premises, smoking cessation counselling and limiting access to tobacco products. The reasons for the limited uptake of the tobacco control role within the community mental health system are complex [2]. Many mental health care providers working in the community lack confidence in their ability to provide smoking cessation counselling; some are ambivalent and may not see themselves as credible role models given their own tobacco dependence [13]. Tailored interventions and close monitoring of clients by health care professionals are often required due to the presence of heavy nicotine dependence in this population combined with the use of psychiatric medications. There are particular issues that require attention when encouraging those with mental illness to quit smoking. People with schizophrenia have been noted to smoke cigarettes to alleviate medication-related side effects such as sedation and neuroleptic-induced Parkinsonism [14]. Tobacco use also affects the metabolism of certain antipsychotic medications (e.g. clozapine and olanzapine) by reducing the serum concentrations by as much as 40% [14,15].

Over the last few decades, the mental health care system has undergone significant changes in the delivery of care. A body of literature examines the roles and perceptions of mental health care professionals within the context of deinstitutionalization [6,16,17]. Research findings point to the overlap in professional roles among providers as well as the gaps in the delivery of services. Some of this research focuses specifically on the required roles of individual professional groups [6] while other studies explore the growing trend of team approaches in community mental health, collaborative delivery of care and shared leadership [17,18]. Less attention, however, has been paid to the role of non-medical providers working in mental health settings [19]. Mitchell's study reveals how some non-medical providers' wish to expand their role within the system. Of note, no literature was found that explored the potential for a shared role in the area of tobacco control among mental health care providers which points to the need for further investigation.

Some qualitative studies have shed light on the smoking behaviours of community-based clients with mental illness, ways to incorporate evidence based tobacco cessation interventions to the unique challenges of people living with mental illness and the barriers and opportunities to the changing culture of mental health settings [20-22]. A qualitative approach is well suited to further exploring how health care providers working in community mental health construct their roles in the domain of client tobacco control.

In this paper, we explore the perceptions of health care providers, both professionals and paraprofessionals, in relation to their roles in tobacco control in the community mental health system. These perceptions are informed by powerful discourses that structure the way the providers think and act. Our particular interest was an examination of the discourses that underlie role perceptions related to smoking cessation intervention. Our approach was based on the assumption that if practices are to be changed, they must first be understood. Discourse analysis has been used to study practices in a wide range of community-based and hospital settings [23,24]. In the field of mental health, Mitchell utilized discourse analysis to examine how mental health care providers in non-medical primary health and social care services viewed their roles [25]. Findings revealed that roles were constructed in opposition to the putative, or assumed role positions of specialist mental health services which the author suggests may contribute to some providers' reluctance to engage with certain agendas promoted by mental health services. A lack of attention has been given to how discourses can play a pivotal role in supporting tobacco control efforts in the translation of health knowledge, which was the impetus for this study.

## Methods

As part of a larger action research project, we conducted a qualitative study focused on understanding the ways that community mental health care providers situate their work related to engaging in smoking cessation support. We sought to understand the discourses that informed how they described their practices. The objectives of the larger project focused on designing and implementing tobacco reduction interventions for uptake by persons working within community mental health systems. For this larger project we targeted six sites. Key staff at each site were identified to collaborate with the project researchers. Using appreciative inquiry [26] that builds on the strengths and priorities of each site, we developed site-specific strategies which would further a tobacco control agenda. As a first step in this study, we conducted interviews with the care providers in the target settings.

Our research approach drew on the methods of discourse analysis. Discourses can be thought of as the sets of common assumptions, values, attitudes, and "rules" that structure the ways in which we think and act [25]. Cheek [27] describes discourses "as the scaffolds of discursive frameworks which order reality in a certain way" (p.1142). These frameworks are often invisible or taken for granted but can be "read" in the ways in which we talk and act. Discourses both enable and constrain the production of knowledge; they allow for certain ways of thinking about reality while excluding others [27]. In this study, we were interested in understanding the discourses that shaped discussion about tobacco control policy and related practices within the community mental health system. Our approach was informed by a Foucauldian perspective in that we sought to understand how discourses operate in ways that privilege certain positions and marginalize or even exclude others. A central concern of Foucault's work is how discourses are shaped and become forms of power that enable particular understandings.

This study took place as a new policy was being introduced that restricted tobacco smoking inside community-based mental health facilities and on their grounds. This policy had been implemented without wide consultation and the health care providers we interviewed were in the midst of adapting to or at times resisting the policy. In addition, those we interviewed had a variety of roles and education; some providers were in paraprofessional roles while others were in professional roles such as nursing, medicine, or occupational therapy. We chose to pool the data paying careful attention to the ways in which power and privilege influences discursive frames.

Data collection occurred in six study locations within the health services district of a large urban setting in western Canada: two community mental health teams, two community resource centres and two mental health housing units. Services at the mental health teams cover assessment and diagnosis, case and medication management, counselling, and psychosocial rehabilitation. The community resource centres provide drop in services to a range of clientele, some of whom are at high risk for other health issues (i.e., homelessness, substance use). These centres are managed by contracted non profit organizations; outreach and social support workers offer services such as crisis intervention, meals, education and leisure programming, advocacy services, life skills, and outreach services. The services offered at housing units vary according to the residents' needs; social service and community support outreach workers provide medication management, case management, support groups, and meal programs.

Data was collected over a period of four months (January-April 2009). Ethical approval was obtained from the relevant ethical review boards. Three research interviewers administered a needs assessment that included quantitative and qualitative data collection. The quantitative component at the beginning of the interview assessed knowledge, beliefs, and practices related to tobacco control and the results will be reported elsewhere. This paper focuses on the qualitative portion of the interview in which the participants were asked to expand on three broad open-ended questions related to: 1) the main issues surrounding smoking in their workplace, 2) their challenges with addressing tobacco use in the workplace and, 3) the types of support, policies or resources that they would find most useful in supporting client smoking reduction or cessation attempts. Initially the interviews were not recorded and detailed notes were kept. However, when the research team realized that the participants had a great deal to share, the decision was made to record the qualitative portion of the interviews. Ethical approval for recording interviews had already been obtained for the larger study. Research staff assured participants that interviews were confidential and that data would be reviewed only by the research team. In total, 79 of the 91 interviews were digitally recorded. Detailed notes captured verbatim content from interviews that were not recorded. The recorded interviews and the notes were transcribed and analyzed by the research team.

The interview data were analyzed discursively by asking questions such as: what assumptions underlie what is being said about tobacco? We were particularly interested in why providers said certain things about addressing tobacco, why they did not say other things and why they selected certain words. To that end, the texts were "interrogated to uncover the unspoken and unstated assumptions implicit within them that have shaped the very form of the text in the first place" [27] (p. 1145).

Recognizing that any text will only ever convey or produce a partial perspective of reality, we were concerned with over interpreting what was said. To prevent imposing excessive meaning, the team met regularly to discuss the development of the discourse analysis. In addition, we embraced the understanding that discourse analysis refers to situated reality. Therefore we considered texts as constructed by and in turn constructing understandings of reality rather than describing *a *or *the *reality [27]. In other words, the ways in which providers talk about their practice reinforces and shapes how they provide care.

As noted, the sample included professionals [n = 42] and paraprofessionals [n = 49]. Over half (63%) of the total sample was female. The average time spent working in the mental health system was 10.3 years and the average time in the current workplace was 4.8 years. Of the 91 participants, 52 were non smokers, 18 were former smokers, 6 were occasional smokers and 15 identified as current smokers. Just under half (45%) of the participants had previously attended some form of training related to tobacco. The sample included frontline staff who worked full time and part time with an adult care population, but not those who worked on a casual basis.

## Results

Our analysis revealed five discursive frames that influenced talk about tobacco. Although other discourses were present in some interviews, the research team was struck by the prevailing presence of these five frames.

### Managing a smoke free environment

As a result of the recent policy restricting where clients could smoke, some providers had assumed new responsibilities. The tasks associated with managing a smoke free environment were framed as placing providers in an authoritarian role. This discourse was most common among the paraprofessionals working in the resource centres and the housing facilities who had acquired the added responsibility of implementing the "new rules."

Investing time in monitoring the smoke-free environment was commonly discussed by resource centre staff. The power of this discourse was evident as they described being involved in crowd control, or "moving people along" when clients were smoking in front of the building. Patrolling was a regular activity because clients resisted the "rules."

*We are constantly going outside and telling people to please smoke in the back. People know that's the rule but they do it anyway. And if you go ask them to leave, they will leave but they won't really stop*.  [Paraprofessional, Non-Smoker, Resource Centre]

The management of the smoke-free environment also encompassed "maintaining the calm" beyond the physical space of the smoke-free setting. Paraprofessionals across settings saw themselves as responsible for minimizing the policy's impact on the local surroundings, referred to by some as the "good neighbour policy." Given that clients were no longer "allowed" to smoke in front of the resource centre, some had moved their tobacco use in front of nearby businesses. As a result, some providers described being further burdened with making amends when there was discord with neighbours.

*So now we have an overflow and the businesses are shooing our members away from their front steps...it's just that they are losing business as a result of the smoking...We are trying to use a lot of tact, and diplomacy and encouragement to be respectful to the businesses and to the members*. [Paraprofessional, Non-Smoker, Resource Centre]

Another paraprofessional conveyed her discomfort with being caught in the middle of managing conflicts: "*We get yelled at by the neighbours and clients." *Such discordance affected the work environment and hampered staff members' abilities to engage with other clients.

For some paraprofessionals there were expressions of resignation, having to "*enforce rules imposed by the city*." The so-called "edict" had been made elsewhere which placed providers in an awkward position vis-a-vis their clients, a position of power and powerlessness. The terminology in this discourse reinforced that this expanded role was a source of tension and "conflict" within the staff-client relationship. According to one paraprofessional administrator, *"It's just a perception that the smoking ban, especially for the chronic smokers, has been kind of forced down everyone's throat. So now we have been asked to police it." *This "*tedious*" responsibility required repeating the same message wherein paraprofessionals had become the target of blame.

*The most challenging for me is having to ask the same people over and over and reminding them of the rules, to step away from the building. They don't even have to say a word, but just the way they are looking at me is kind of like I have come up with the rule*. [Paraprofessional, Current Smoker, Resource Centre]

As self-declared managers of the smoke-free environment, some staff positioned themselves as embroiled in uncomfortable power dynamics. When clients ignored the "rules" by smoking within smoke-free zones, paraprofessionals were obliged to exert control and impose "penalties." At the resource centres, this could mean banning clients for two days, a serious consequence for clients who relied heavily on the services, *"that means that they don't get the service, they don't get lunch."*

Resistance was a common element in this discourse and took the form of not always enforcing the "rules." For some, priority was given to the clients' well-being and rules were bent accordingly, claiming this was more in keeping with the perceived role of a service provider. Circumventing rules was a way to avoid conflict in the client-provider relationship.

*I would lie if I said that we strictly enforce the 3-meters-from-a-door deal. I'm not going to make somebody who is having a bad day go stand in the cold rain. If they're going to stand under the awning out the back door, it's fine because the door is always closed, the smoke doesn't come in anyways*. [Paraprofessional, Current Smoker, Resource Centre]

Similarly in one residential facility, staff would not "*rat out*" the clients for smoking in undesignated areas; no staff member wanted a client to be fined $2000, all the while recognizing the futility knowing that the Health Authority would end up "*paying for it*."

On the mental health teams, the discourse was much more subdued. The smoke-free environment was a better fit among the multidisciplinary teams than it was at the resource centres and residential settings where more providers were smokers. Nonetheless, there were hints of resistance with suggestions of inconsistent action. As one professional noted, "*Sometimes those regulations are transgressed by clients and some staff will approach the person and correct them, and some won't." *Given that monitoring the smoke-free environment was a new "*duty*," there were now "*watchdogs*" on the teams. On rare occasions, "*relocating*" clients was necessary; that became the responsibility of "*someone else*," typically a senior staff member which provided some professionals a safe distance from dealing directly with the issue.

*There have been some conflicts with clients smoking right outside our facility, despite the signs. There was almost an altercation when a staff asked a client to stop smoking right in front and go down the stairs*. [Professional, Non-Smoker, Mental Health Team]

Interestingly, the professionals on the mental health teams maintained that they were critical of punitive measures. This stance was perhaps easy to take given that they did not need to take such measures.

### Tobacco is therapeutic

The discourse that smoking "*helped*" clients was present in interviews across all settings. At the root of this discourse was the claim that tobacco use was not only beneficial, but that quitting smoking was difficult and potentially harmful for clients. Tobacco use was described as providing relief from symptoms associated with mental illness. There was a common conviction that tobacco use countered some side effects of "anti-psychotic" medications, therefore *"It helps them with their medications to be able to have their cigarette." *Knowledge derived from work experience pervaded this discourse.

*We know that somehow tobacco use helps schizophrenia or psychiatric clients to cope with their symptoms. And the years of observation of the clients in the hospital or during any activities here in this setting, obviously it has a calming effect, or at least they are severely hooked on that*. [Professional, Former Smoker, Mental Health Team]

Others considered cigarettes to be a "*quick fix*" and "*instant pleasure*." The language focused on the positive effects of smoking in keeping with self-medication terminology. Tobacco was lauded for providing the clients' *"only joy in life." *The comparison of tobacco to other substances served to minimize the harmful effects of tobacco. One paraprofessional challenged, "*I question the effects of smoking compared to the effects of prescription medications that they are taking, compared to the coffee that they are drinking, everything else in their lives*."

As further support, the providers emphasized the dangers of quitting smoking, placing clients at risk. One professional elaborated, "*At one point, there was a client who attempted to end his smoking but he became so stressed and it started to impact his mental health." *Tobacco cessation was framed as removing "*their comfort*" which reinforced that smoking was beneficial and served a purpose.

*For some, smoking is a core part of their stress coping. And for some I think it is really important to continue smoking because that is all they have...For some, taking away their cigarettes is the worst possible thing you could do to them*. [Professional, Former Smoker, Mental Health Team]

The strategy of minimizing the therapeutic value of other forms of nicotine infused this discourse. Nicotine replacement therapies did not "*work*," and they were costly and problematic. "*And a lot of people can't wear the patch for allergies, can't chew gum because of dental work." *Another provider concluded that social support was absent when using these forms of nicotine, cautioning that clients could become "isolated", "*There is nothing social about NRT, you get together and have a coffee and cigarette. You don't get together to chew gum." *In contrast, the social role of cigarettes was presented as beneficial for clients. "*Even outside of the actual addiction to the nicotine, it's also the addiction to the smoking to having the relationships that they do. People get into patterns of smoking out there with particular people, having particular types of conversations."*

Smoking with clients outdoors functioned as a therapeutic tool; some providers who smoked positioned this action favourably. *"Often it's a way to just get someone to calm down too. 'C'mon, let's just go out there and relax and have a smoke.'" *The shared activity was described as a conduit for relationship building, suggesting a privileged relationship that was also power-laden in that it maintained the status quo.

*I've seen it as being beneficial in a sense since you have that time where you sit down, even though it's just for a cigarette, you have that one-on-one interaction. So in that sense, I've developed a lot of trust from going out there and just having a casual conversation that I wouldn't have had within the building surrounded by people*. [Paraprofessional, Current Smoker, Resource Centre]

One staff member had quit smoking 6 months earlier and spoke with nostalgia about the strength and value of the client-staff connection through smoking and how the intimacy that existed was now "*lost*." Not only did she feel more "*in touch*" with what was going on with clients, smoking with clients also functioned as an opportunity for information sharing.

*A lot of the work I was doing came from...sitting down with people and smoking and talking about stuff...they would kind of relax a little bit and we'd get talking....I miss that connecting with people on that level*. [Paraprofessional, Former Smoker, Resource Centre]

Nonetheless, a sense of discomfort was articulated by some providers. One provider "*struggled*" with his role in this shared activity "*we are trying to assist people to quit smoking, but we smoke with them."*

Descriptions of accommodating client requests for cigarettes and rolling paper was part of the therapeutic discourse. *"I'll give them the end of a cigarette, but I won't take it back. I just don't, you never know what they might have picked up or germs or things like that." *The availability of rolling paper at the resource centres was framed as meeting a need and providing a healthy alternative that counterbalanced the health risks associated with clients' practice of "picking up butts."

### Tobacco use is an Individual Choice

The discourse that framed tobacco use as an individual choice focused on the autonomy of clients and how in relation to tobacco use, they "*have the right to choose." *By engaging with clients in a manner described as "respectful," clients were presented as in charge and responsible for deciding about their tobacco use in discussions that took place within the context of following the client's lead.

At the outset, this discourse revealed a distancing strategy. Providers described a reluctance to formally initiate the topic of smoking with clients. Some explained how they did not assess client smoking when they first met because it would seem "*judgmental*." They reasoned that assessing tobacco use at the initial assessment had not been a part of their training. Other goals took precedence for both clients and, subsequently staff.

*When I first work with people I ask them what their goals are, what things they would like to achieve in addition to seeing us for mental health symptom management... I usually take it as a cue from them when they feel as if they are ready to engage in discussion about smoking cessation, but for sure, it is not high on my priority list. And I certainly don't see it very high on some of my client's priority list either*. [Professional, Non Smoker, Mental Health Team]

Defending a position of "*not pushing*" or even omitting the smoking cessation agenda altogether was common. Ultimately, clients were placed in a position of power, being in charge when it came to addressing tobacco use.

*I find a lot of the clients, if it is brought up, it's just brushed off or not discussed in depth. And so if I mention it or bring up smoking, then they're just "Oh yeah, that's not an issue," then I'll drop the issue as well*. [Professional, Non-Smoker, Mental Health Team]

Silence regarding client tobacco use was framed within the context of being respectful, *"If they are not open to having the conversation, then I don't usually pursue it." *Viewed from another angle, this discourse served to justify providers' inactive engagement. Although labelled as showing "*respect*," this meant no further action on the part of the provider. The language used was moralistic and prescriptive. One professional concluded, *"I see it as people's choice, especially in the community people are able to make their own choices and be responsible for themselves, be autonomous for their own care."*

Treading lightly was a tactic employed to avoid discord while supporting clients' right to smoke. Some providers expressed uncertainty, discomfort and a sense of "*struggle*" when it came to addressing tobacco use with their clients. As one psychiatrist noted, "*I admit that I don't address it enough...I still have the hesitancy about how to work it in there and how to not make it something that is just another burden to patients." *In fact, staff-driven attempts at initiating a discussion about smoking cessation were described as "*hassling*" clients about "*one more thing*."

*If they haven't really expressed any idea about quitting, how to bring it up in a way that won't turn them off? Because that's what I feel that right away they retreat from that approach. If it starts to put them off, right away you are going to lose them*. [Paraprofessional, Non-Smoker, Mental Health Team]

Consequently, honouring clients' lack of interest regarding the topic of smoking cessation allowed the client to exercise their unchallenged choice to continue to smoke.

The responsibility and "*choice*" of remaining a smoker was placed into the hands of clients. Clients were described as "*reluctant to quit*," and lacking "*motivation*." Speaking knowingly about the power of clients' nicotine addiction further removed the onus of personally engaging with clients and served as a distancing device, "*They would rather have a cigarette than food. These people are addicted to tobacco. This is a very strong addiction - they cannot control themselves with nicotine." *However, offering clients "*information*" such as brochures about smoking cessation programs was considered a non intrusive gesture that allowed clients to *"make a choice about it."*

In contrast, client-led invitations to engage in the topic of smoking were met with enthusiasm. These opportunities occurred when the client-provider relationship was well established. *"When somebody talks about smoking, I leap on it and I say, 'The Daytox, they have an excellent program.' And I'm excited and I really, really encourage people once they articulate it." *Such interactions were presented as the moment for planting a favourable seed that might influence a client's choice about tobacco use in the future, conveying a glimmer of hope.

### It's Someone Else's Role

In this discourse, providers dismissed the role of directly supporting client tobacco cessation. Rather, they framed this role as belonging to an "*expert*." Relying on available resources such as support groups or other professionals with expertise area figured prominently in their explanations.

Both paraprofessionals and professionals viewed the scope of their role in smoking cessation to be limited. A staff member from one housing facility stated, "*it's not necessarily our role to dictate to people what they should be doing with their lives." *There was often a shift of focus when providers looked to others to assume the role. Some professionals saw this as a specialized skill set beyond their domain while some paraprofessionals viewed the professionals as the experts.

*Perhaps an in-house therapist/counsellor can be accessed. I don't think it's that much a physician needs to be involved in. Identify the problem and encourage them to quit, yes, but in actually providing cessation counselling and that type of stuff, I don't think so*. [Professional, Non-Smoker, Mental Health Team]

*I am not the one who can control her *[client] *smoking, her attitude or her routine, but the nurse and the psychiatrist can do that, I'm just their worker. I just tell them that smoking is harmful to your health*. [Paraprofessional, Non-Smoker, Mental Health Team]

Typically, this discourse involved accentuating a lack of training and knowledge regarding tobacco use. As one paraprofessional surmised, "*I am comfortable addressing and looking at readiness in terms of quitting smoking but personally, I don't know that much about implementing smoking cessation goals." *At times, the role of engaging with clients in tobacco cessation was presented as unattainable.

*It's an addiction, so you need a lot of resources to help out with it. It would never be something that I could do. It's not like something else where I could help them, like by referring them to a job*. [Professional, Non-Smoker, Mental Health Team]

Multiple-roles and conflicting priorities were offered as reasons for not being able to assume this role. This strategy maintained the comfortable power and position of the health care provider yet ceded specialized knowledge and power to others. For one professional, taking on the issue of tobacco use was portrayed as adding to the "*workload*" in an already "*overwhelming*" multidisciplinary team environment. Another professional expanded, *"It's another thing to consider because at the same time we are dealing with diabetes because that is a huge concern for our clients. We are taking on that whole metabolic monitoring and now the smoking as well." *Being a smoker was sometimes used as a rationale for dismissing the role entirely.

*As I am right now in the middle of trying to stop, I just don't find that it makes sense for me to say things about smoking when I am a smoker, inside I would feel guilty. And so say to them it's not good to smoke and this is what you should do*. [Paraprofessional, Current Smoker, Resource Centre]

To ease the tension of not engaging in this role, distributing educational brochures about tobacco use and cessation programs filled a useful function - providers had satisfied their responsibilities. This also strengthened the message that others had tobacco cessation expertise, reinforcing that this responsibility belonged elsewhere. In the same way, referring a client elsewhere for tobacco cessation support was presented as being professionally responsible; this specialized role belonged to someone else. It was, however, safe territory to promote the advantages associated with joining these programs, *"So if I know they smoke, I will talk to them about this program, this free patch and free nicotine and I encourage them to join."*

### Tobacco Control as Health Promotion

In the discourse of health promotion, tobacco was powerfully described as "*unhealthy*," a "*drug*" that was "*highly addictive*" and linked with cancer and cardiovascular complications. Many providers recalled clients and their own family members who had died from tobacco-related causes. This health-focused discourse translated into promoting tobacco cessation, and harm reduction approaches, therefore challenging the assumptions of the previous four discourses. Formal interventions belonged to professionals who had the expertise while paraprofessionals engaged in an informal discourse, encouraging and reinforcing the message, which often highlighted a personal style.

Cessation interventions by professionals were characterized by a specialized skill set involving counselling (e.g. motivational interviewing) and pharmacological interventions. Some descriptions focused on the human element of engaging, others on the bio-physical aspects of treating the addiction; both conveyed that there was an expert and voice of authority.

*And if they are motivated about it, I can engage them in a process of discussion and interest. I have some sense of the tools available and so on. It's very satisfying. You know when I was in general practice and I would help people quit smoking, years later when I would see those people... they would say 'You're the one who helped me quit!' *[Professional, Former Smoker, Mental Health Team]

Weighing competing risk factors and tailoring approaches accordingly were key elements of interventions. The health promotion discourse revealed that certain clients received more attention about smoking cessation than did others, specifically, those with other medical conditions. One professional prioritized his client's "bad airways," *"I have a young guy*...*He also has terribly severe asthma and he's really pre-contemplative about changing his smoking. But I've spent a lot of time trying to move him along to that stage of change."*

The content and the delivery of the health promotion message varied according to their clients' socioeconomic status and the perceived level of function. The expert provider was in a position to know how the message would be interpreted.

*A lot of our clients, they are really poor. They have no stimulation in their life. And I see cigarettes as really important to them...For some, it's taking a core part of their identity away, their best friend away. So for those, I will have a different approach, or a softer approach*. [Professional, Former Smoker, Mental Health Team]

The timing of targeted interventions was important. This meant that the client had to be "*stable*" and it had the tone of the expert taking charge when timing was "appropriate" when the client was "activated in their recovery process, as exemplified by one professional *"I address it more as 'Oh, you are really doing well, you are more stable, you are making your appointments, you are taking your meds, your symptoms aren't bothersome now, why don't we look at dealing with the smoking issue?’” *Knowing when it was the "right time" was portrayed as a skill, with the emphasis placed on holistic well-being. According to one professional, "*To me it seems like an art, experiential, knowing when to push and when to back off, how to rate it among people's other issues, looking at the whole picture."*

Intervening to reverse tobacco reduction or cessation was described as the necessary harm reduction measure when the timing was not right for a client's "*mental health*." In this situation, the expert voice of authority ruled, *"I advised her to forget it now because she is not stable."*

Some providers described how they looked for and worked with "*outward signs*" when clients were "*coughing like mad*." Such moments were considered to be a "*good time*" to raise the topic of tobacco use: "*I'm a nurse, so if someone is coughing a lot, I will say 'So how much are you smoking and do ever think of cutting back?'" *Another provider recalled transforming a situation of being "*on the spot*" during a shopping assessment into an opportunity to engage in tobacco cessation discussion.

*It is awkward because they say, "Do you mind if I have a cigarette?" and I kind of do... I asked him if he had smoked for a long time, would he be interested in [quitting] smoking. He said "Maybe one day." At the end, I gave him the information about the ButtOut group. It was an opening*. [Professional, Non-Smoker, Mental Health Team]

There was a call for immediate intervention when client smoking posed a serious safety concern as was the case for one professional whose client, a woman with alcohol dementia, had caused a fire in her apartment. "*I'm really working very hard to get her to stop smoking because not only is she at risk, but so are the people in her building." *This was further described as "*beyond an individual's right to do something that is unhealthy*" and more about protecting others from "*physical harm*."

An informal harm reduction approach was a part of the health promotion discourse. One paraprofessional claimed to not "*have any information*" for her clients, but reminded them of the health and financial benefits of cutting back. Another provider explained, "*I encourage more of a harm reduction approach. I see a lot of people do the cold turkey thing and come back to it and I expect there's guilt." *Those who had been personally touched by the tobacco-related loss of a family member dedicated themselves to fully engaging in this discourse.

*I myself am not a smoker but I do know it's addictive, I do know people go through a pack a day, so I just try to start conversations, "Oh, how much do you smoke? Oh, a pack a day. Okay, what are you hoping to do, like next month? Are you hoping to reduce? What's another thing that you could substitute it with? *[Paraprofessional, Non-Smoker, Resource Centre]

Attentive communication was emphasized in the health promotion discourse. "*So I have to listen to what their plan is and reflect it back. And hopefully they have time to think, 'Oh, is it a reasonable plan?'" *Although continual efforts to engage clients were labelled "*frustrating*" the potential for tobacco reduction or cessation was held up as an option: *"They say, 'I cannot quit smoking, I am addicted. I've smoked for fifty years or twenty years, for many years.' And I say, 'Can't you just cut back a little bit?'" *Trying to "*grasp at something*" was a device used to keep the tobacco discussion open with one client who was grieving the loss of her son "*She has one remaining son. That son has now had a son and I say "Don't you want to be around to see that grandson?"*

Personalised approaches were adopted when exploring client motivation to quit smoking. One paraprofessional spoke with confidence about investigating client motivation by "*testing readiness*" in a way that resembled a formal approach.

*You recognize patterns and human behaviour and if it's not a level of motivation, something that I would grade over a 7, I wouldn't invest a lot of time supporting that. So I am looking and testing levels of motivation whether it's through body gestures, eye contact, things that they are saying, if they've done their research on their options*. [Paraprofessional, Current Smoker, Resource Centre]

Another paraprofessional, a smoker, was adamant that he would "*never stop*" talking to his clients about smoking cessation, albeit his words were flavoured with wishful thinking. *"I hope that one day when they are on about it, I happen to say the right word, the right combination of words and just catch them at that right moment where it's going to work."*

There was tension beneath the language of this discourse. Approaching the topic of tobacco use was a "sensitive" matter that called for self reflection. One professional wrestled, "*You have to ask, is this respectful, am I preaching to them, am I judging them? So it's a really delicate balance to play with smoking." *Nevertheless, there was relief with the recognition that some clients had been successful in their efforts to quit smoking.

*We used to be afraid to bring it up, because that would destabilize people, they can't handle it, but now we know it can be hard but with the right support, people can actually reduce or quit their smoking. So that is a tremendous shift in attitudes*. [Professional, Non-Smoker, Mental Health Team]

## Discussion

This study is not without limitations. We acknowledge that we do not capture all of the discourses related to tobacco control in community mental health settings. Nor do we claim that the discourses reported are taken up by mental health providers in other settings. The analysis does however provide useful insights into the relationship between organizational structures and provider practices.

Although there was a discourse that clearly dominated each one of the provider interviews, the providers often invoked multiple discourses during the course of the interview. Typically, the dominant discourse appeared alongside a secondary and at times tertiary discourse that backed up the main discourse. For example, a central discourse of tobacco as "therapeutic" was often accompanied by the minor discourse of "individual choice" with some minimal reference to the discourse of "managing a smoke free environment" in the background. This movement between discursive frames may have been a reflection of the transition and state of flux within the mental health care system with the introduction of the new smoking policy.

The presentation of the discourses is not intended to portray community mental health care providers in a negative light; rather, our aim is to highlight the assumptions of those working within a climate of recent organizational change. The providers in this study were indeed concerned about their clients' well being, albeit, how this was manifested in terms of tobacco control practices varied widely. Importantly, the findings highlight the challenging context within which many community mental health care providers are working and within which smoke free policy is being attempted and therefore point to direction for future action. For example, the discourses of tobacco use as "therapeutic" and "an individual choice" point directly at systemic obstacles to changing practice and the "historical situatedness" of tobacco in the mental health system. Of note, these two discourses mirror the findings in the Lawn et al. 2002 qualitative study of community-living clients who described smoking as a way of finding companionship and sense of identity while cigarettes themselves were a symbol of control [20]. There are undoubtedly ongoing opportunities to challenge and shift some providers' beliefs about "the power" of tobacco for their clients, for example the common interpretation that tobacco use serves as a tool for "intimacy" within the client-provider relationship [20]. Approaches to practice change need to take into account these embedded perspectives. As Waring and Currie [28] point out, practice change is rarely best accomplished through a "top-down" approach; in their words, "to 'harvest' best practice through learning requires an approach that allows for customization and localization" (p. 775).

The discourse of "managing the smoke-free environment" is particularly interesting as it exemplifies the complex and interdependent relationship between structure and agency. It is tempting to conceptualize policies as objective and external to health care providers, yet it is the actions of the providers that make these policies "come alive" and enforce their use. The providers in this study embodied the new tobacco policy in their actions even though many wanted to resist it. Indeed, rather than questioning the policy, or determining how it could best be implemented, many took up the role of enforcing it, thus socially and discursively constructing the tobacco policy. From a Foucauldian perspective, this discourse provided the mechanism to ensure that providers maintained their own power within the system. Many of the providers reinforced the rules because they were at a loss as to what else they could do. As Davies and Nutley remind us, organizational change cannot easily be wrought by simple exhortation [29]. Successful strategies need to take into account the needs, fears, and motivations of staff at all levels. Furthermore, any attempt to influence key practices must be part of a much wider approach that addresses concerns related to resources, power and control.

The issues of power are also interesting to consider in relation to the discourse of tobacco use as "individual choice." The meaning and value of cigarettes for clients has been well documented and is increasingly recognised [20]. Although the respect of clients' choices is laudable, at the same time it denies the contextual factors at play that reinforce tobacco use among those with mental illness. In fact, this position appears to cede more power, privilege and authority to clients than is warranted, thus absolving the provider of any responsibility for addressing issues of tobacco use. Similarly, the claim that tobacco is therapeutic because it is the client's "only joy" frees the provider from considering ways to assist clients with finding pleasure through other means. Why is it that clients are not provided opportunities for other sources of pleasure? What structural changes are required to help clients meet their needs? While we need to be respectful of clients' choices, it is also important for us to consider how their choices may be constrained by social and structural factors. Poland and colleagues suggest that tobacco control programmes have not sufficiently addressed the issue of power relations and the central importance of context with respect to smoking. If the discourse can be shifted wherein tobacco use is seen as a socially embedded practice, providers may be prompted to consider the ways their own power and social locations influence relations with those who use tobacco [30].

The discourse of "it's someone else's role" may in part be a lingering symptom of the "historical divide" between addiction and mental health services. Unfortunately, the "hands-off" approach implied by this discourse resulted in a gap in services for clients who needed support within the context of the new "smoke free" policy. Goldberg points to the evolution of integrated treatments in which some clinicians (psychiatrists and psychiatric nurses) now recognize their role in medication and symptom monitoring, therefore supporting clients who adopt a smoke free lifestyle [31]. Similarly, our study findings reflect a system that is evolving, one in which some community mental health providers are beginning to rethink their ethical obligations, roles and responsibilities.

The health promotion discourse was taken up predominantly by members of the mental health teams where there was strong support for tobacco control. Nonetheless, this discourse was also present in the other settings despite the fact that those providers were heavily engaged in controlling the location of tobacco use. Some professionals and paraprofessionals made use of openings regarding tobacco reduction or cessation in all settings. Goldberg proposes a shift has already occurred within the mental health system where past missed opportunities are now replaced by supporting and motivating clients in the process of change towards a healthier lifestyle [31]. Certainly the findings of the health promotion discourse convey optimism within a context of change in which providers' use of "openings" and motivational interviewing can contribute toward a smoke-free lifestyle for clients [32].

Our findings add to the limited literature examining the role of community mental health providers in the domain of tobacco control and clarify direction for continued efforts. Despite some brief training for staff at the introduction of the smoke free policy, knowledge and skills' deficits regarding the implementation of tobacco control measures were widespread. As a result, some providers were unprepared for the policy and did not have the tools to advocate for practice or policy changes. Staff training and ongoing educational needs must be incorporated, paying attention to realistic expectations of the work environment as well as the overall culture, dynamics and uniqueness of each setting. Other research supports the benefits of staff training in terms of smoking cessation practices and preparedness [7].

The study findings suggest that providers' own smoking status can affect the ways in which they engage in tobacco related interactions with clients, consistent with other research [4]. Offering cessation support to interested staff members could contribute to a culture of change within the mental health system by promoting "wellness" focused dialogue between providers and clients regarding tobacco use [22]. In addition, educational support to non smoking staff would strengthen their abilities to advocate for smoke free environments. In the spirit of collaboration and appreciative inquiry, we must continue to ask what providers want and need to fulfil their roles [26].

Despite efforts to provide smoking cessation support to people with mental illness, a gap remains between science and service [33]. In the current study, despite key policy changes towards a smoke free environment, there was no systematic uptake of other practice changes. While implementing smoke free policies is an important and timely intervention in itself, the lack of managed change process can unintentionally reinforce negative attitudes about tobacco control initiatives.

The misinformation about tobacco use and mental illness was most pronounced in the discourse of tobacco use as "therapeutic." Similarly, Ziedonis et al. have emphasized the importance of challenging misconceptions about the putative therapeutic benefits of tobacco use which contributes to reluctance on the part of some providers to support reduction or cessation efforts [2]. Ferron et al. point to the controversy surrounding this "self-medication hypothesis," noting that study results have been mixed [14]. Accurate information focused on the safety and efficacy of nicotine replacement therapy and other smoking cessation medications translates into health messaging that can be useful and ethically sound. The concern that quitting smoking is too stressful for individuals with mental illness is not supported by empirical evidence and can therefore be viewed as an opportunity to dispel a long standing myth within this system [8]. Smoking cessation interventions can be effective for some people with schizophrenia and related disorders [14]. The introduction and resourcing of empirically sound health promotion efforts would contribute to shifting tobacco control discourses within the community mental health system. As Ziedonis and colleagues suggest, addressing tobacco use in the context of mental illness requires both program and system level change [34].

## Conclusions

Practice change cannot take place in a vacuum. Rather it must be based on a solid understanding of the contextual factors that shape knowledge, attitudes and practices. This study provides insights into the complex discourses that shape tobacco control practices in the mental health field and reinforces the need to see practice change as a matter of that extends beyond the individual.

## Competing interests

The authors declare that they have no competing interests.

## Authors' contributions

JLL designed the larger study, conceptualized and participated in data analysis, and participated in writing the manuscript. BMM collected and analyzed data, and participated in writing of the manuscript. LAM managed the larger study, collected data and participated in writing of the manuscript. All authors read and approved the final manuscript.
